# Clinical characteristics and outcomes of COVID-19 patients with varying renal functions statuses during the Omicron pandemic in Shanghai

**DOI:** 10.1186/s12882-023-03258-3

**Published:** 2023-08-24

**Authors:** Tian Xu, Zhiyao Bao, Min Zhou, Weiming Wang

**Affiliations:** 1 Department of Nephrology, Ruijin Hospital, Shanghai Jiaotong University School of Medicine, Shanghai, 200025 China; 2 Institute of Nephrology, Shanghai Jiaotong University School of Medicine, Shanghai, 200025 China; 3 Department of Pulmonary and Critical Care Medicine, Ruijin Hospital, Shanghai Jiaotong University School of Medicine, Shanghai, 200025 China; 4 Institute of Respiratory Diseases, Shanghai Jiaotong University School of Medicine, Shanghai, 200025 China; 5 Shanghai Key Laboratory of Emergency Prevention, Diagnosis and Treatment of Respiratory Infectious Diseases, Shanghai, 200025 China

## Abstract

**Objective:**

This study aims to provide an academic summary of the clinical characteristics, outcomes and risk factors associated with prolonged hospital stays among the patients with varying renal function statuses during the Omicron pandemic in Shanghai.

**Methods:**

Clinical data was collected from COVID-19 patients admitted to Shanghai Jiaotong University School of Medicine Ruijin Hospital Northern District. Based on their baseline eGFR, the patients were divided into three groups: Group A (eGFR > = 90ml/min/1.73m^2^, *n* = 384), Group B (15ml/min/1.73m^2^ < = eGFR < 90ml/min/1.73m^2^, *n* = 220), and Group C (Hemodialysis-dependent patient, *n* = 92). Clinical characteristics and laboratory data were compared among the three groups. The cumulative hazards of ICU admission were compared using the Kaplan-Meier method. Univariate and multivariate linear regression analyses were conducted to identify the factors influencing the duration of positive nucleic acid test.

**Results:**

Between March 25, 2022, and May 31, 2022, a total of 696 COVID-19 patients were included in the study. Among the dialysis patients, 92% (85) of dialysis patients had not received any COVID-19 vaccination, and 14.1%(13) of hemodialysis (HD) patients eventually progressed to severe or critical cases. A total of 13 (2.15%) patients were admitted to the ICU, with 8 (61.5%) were HD patients. The duration of nucleic acid positivity showed a negative correlation with eGFR (B: -0.048, 95%CI: -0.059~-0.037, *P* = 0.000), platelet counts (B: -0.011, 95%CI: -0.017~-0.005, *P* = 0.001) or lymphocyte counts (B: -0.658, 95%CI: -1.229~-0.086, *P* = 0.024).

**Conclusions:**

The majority of Omicron patients have a favorable prognosis, while HD patients experience relatively poorer outcomes and higher rates of ICU admission. Decreased eGFR and low lymphocyte/platelet counts are the important risk factors associated with prolonged Omicron infection.

## Introduction

The world has experienced multiple waves of global pandemics since the first reported case of severe acute respiratory syndrome coronavirus (SARS-CoV-2) in November 2019. As the pathogen causing COVID-19, SARS-CoV-2 virus has mutated multiple times since then. One such variant, the Omicron variant, was identified in South Africa in November 2021 and exhibited a higher number of mutations compared to previous strains of the virus [1]. The Omicron variant is known to be more infectious than previous COVID-19 variants [2]. Although this variant increased transmissibility, its morbidity and mortality seems to diminish markedly. Because this variant with a reduced virulence caused only a mild to moderate increase in hospital admissions, a lot of countries loosed or completely cancelled public health measures previously carried out. Consequently, the Omicron variant rapidly spread across the globe. According to the data of World Health Organization (WHO), a second sub-lineage of the Omicron variant (BA.2) began replacing BA.1 in most regions worldwide from March 2022 [3]. From late March 2022 to the end of May 2022, the variant strain caused nearly 650,000 infections and approximately 600 deaths in Shanghai [4].

Chronic kidney disease (CKD) patients always associate with immune activation or deficiency, resulting in systemic inflammation or increased susceptibility to infection. Therefore, early reports of the COVID-19 outbreak had already identified CKD as a risk factor for severe COVID-19 [5]. Another large-scale study including 17 million health records also reported CKD as a risk factor for poor prognosis in SARS-CoV-2 patients, with glomerular filtration rate (GFR) < 30 ml/min/1.73 m^2^ conferring a high risk in multivariate analyses [6]. In particular, the 28-day mortality rate of COVID-19-positive dialysis patients was 21.1 times higher than that of the other dialysis patients [7]. Moreover, end stage renal disease (ESRD) patients often experience poor nutritional status and emerge with more comorbidities, resulting in significantly elevated mortality rates compared to the general population. However, the relationship between the Omicron variants with reduced virulence and CKD, especially ESRD, remains unclear.

This wave of pandemic occurred after most of the people were widely vaccinated in China. Our primary concern is to identify which patients are at risk of developing clinical symptoms or progressing to severe cases. Therefore, the aim of this retrospective study was to summarize the clinical characteristics, outcomes, and risk factors associated with prolonged hospital stays among patients with varying renal function statuses during the pandemic in Shanghai.

## Methods

### Study design and setting

This retrospective, observational study was conducted at Shanghai Jiaotong University School of Medicine Ruijin Hospital Northern District, which was completely converted to COVID-19 dedicated hospital following the pandemic outbreak. The study was approved by the Independent Scientific Advisory Committee of the Shanghai Jiaotong University School of Medicine Ruijin Hospital (Approval Number: 2022078). The study was conducted in accordance with the principles outlined in the Declaration of Helsinki. This study population included 696 patients who were admitted to the hospital with Sars-Cov2 positive RT-PCR nasopharyngeal swab between March 25, 2022, and May 31, 2022. A multidisciplinary team consisting of pneumologists, nephrologists, infectious disease specialists, emergency care physicians and anesthetists was involved in the management and care of the patients.

All data utilized in the study were collected from the electronic medical records prior to analysis. The requirement for written informed consent was waived by the Ruijin Hospital Ethics Committee due to the retrospective nature of the study and the high biological hazard.

### Participants

This study included patients aged 18 years or older who had positive results for SARS-CoV-2 on RT-PCR nasopharyngeal swabs, with at least two consecutive positive results.

### Data collection

Upon admission to the hospital, comprehensive baseline clinical characteristics of the patients were collected, including age, gender, COVID-19 symptoms, vaccination status, and comorbidities such as cardiovascular disease (CVD), hypertension, diabetes, malignancies. Additionally, initial laboratory data were recorded, encompassing serum creatinine (sCr), blood urea nitrogen(BUN), albumin, electrolyte levels, transaminase levels, blood cell counts. Medication information, including the use of antibiotics and Paxlovid, was also documented. Based on baseline sCr values, estimated glomerular filtration rates (eGFR) were calculated. Futhermore, the dates of admission, discharge, transfer to intensive care unit(ICU) were collected for each patients.

### Definitions

CVD was defined in this study as the presence of any of the following conditions: coronary heart disease, acute or chronic heart failure, arrhythmia, cardiomyopathy, heart valve disease, or stroke. eGFR was calculated using the EPI formula. According to the guideline [8], the severity of COVID-19 was determined based on established criteria. Mild cases were defined as patients who had mild clinical symptoms (fever and/or respiratory system symptoms, etc.) without sign of viral pneumonia on chest imaging findings. Moderate cases were defined as patients who had symptoms along with sign of viral pneumonia on chest imaging findings. Severe cases referred to patients who met any of the following criteria: respiratory rate > = 30 breaths/min; oxygen saturation < = 93% at rest state; arterial PO_2_/oxygen concentration < = 300 mmHg; pulmonary lesion progression > 50% within 24–48 h on radiologic imaging. Critical cases were related to patients who met any of the following criteria: respiratory failure needing mechanical ventilation; shock; transferred to ICU due to multiple-organ dysfunction.

### Follow-up and outcomes

The follow-up period was from the date of hospitalization until the day of discharge or admission to the ICU. The length of hospital stay for each patient was meticulously recorded. The study outcomes of interest included the occurrence of ICU admission and the duration of positive nucleic acid test results.

### Grouping

Based on their baseline eGFR, the patients in this study were classified into three groups: Group A (eGFR > = 90ml/min/1.73m^2^), Group B (15ml/min/1.73m^2^ < = eGFR < 90ml/min/1.73m^2^), and Group C (Hemodialysis-dependent patient).

### Statistical methods

Data analysis was conducted using SPSS 22.0 statistical software (IBM Corp: Armonk, NY, USA, 2013). Statistical significance was defined as *p* < 0.05. Categorical variables were expressed as frequency (n) and percentage (%). Normally distributed quantitative variables were expressed as mean ± standard deviation, while non-normally distributed quantitative variables were expressed as median and interquartile range (IQR). Chi-square tests were performed to compare categorical variables between the groups. For normally distributed variables, one-way analysis of variance (ANOVA) was used, and pairwise comparisons were performed using the least significant difference (LSD) method. Non-normally distributed variables were compared using the Kruskal-Wallis H-test. The cumulative hazards of admission to ICU among the groups were assessed using the Kaplan-Meier method. Univariate and multivariate linear regression analysis were conducted to identify the factors influencing the duration of positive nucleic acid test.

## Results

### Clinical characteristics and laboratory data of patients with COVID-19

Between March 25, 2022 and May 31, 2022, a total of 696 COVID-19 patients were included, with an average follow-up time of 13.99 ± 5.98 days. Among the dialysis-dependent patients, the prevalence rates of hypertension, diabetes, CVD, and malignancies were significantly higher compared to the other two groups. Notably, over 92% of dialysis patients didn’t receive any COVID-19 vaccination, whereas 444 (73.5%) patients in the other two groups received at least 2 COVID-19 vaccine shots.

Although no significant differences were observed in the symptoms among the three groups, severe/critical cases were more prevalent in the dialysis-dependent patients. The majority of patients with normal eGFR and non-dialysis dependent CKD patients were asymptomatic or mild cases. In contrast, 14.1% of dialysis-dependent patients eventually progressed to severe or critical cases. Most of these patients received symptomatic and supportive treatment, while only 49 cases (7%) underwent oral Paxlovid therapy.

Furthermore, in addition to having poor nutritional status (lower albumin and hemoglobin levels) and electrolyte imbalance (higher potassium and phosphorus levels), dialysis patients exhibited lower transaminase levels and reduced counts of neutrophil, lymphocyte, platelet (Table 1).

**Table 1:** Clinical characteristics and laboratory data of patients with COVID-19

	Group A *n* = 384	Group B *n* = 220	Group C *n* = 92	*P value*
Age (y)	41.21 ± 14.44^#^	59.30 ± 15.0^#^	64.57 ± 12.81	0.000
Male (n, %)	219, 57.0%	126, 57.3%	57, 61.9%	> 0.05
Comorbidities				
Hypertension (n,%)	34, 8.9%*	75, 34.1%*	73, 79.4%	0.000
Diabetes (n,%)	15, 3.9%*	26, 11.8%*	30, 32.6%	0.000
CVD (n,%)	2, 0.5%*	22, 10%	17, 18.5%	0.000
Malignancies (n,%)	2, 0.5%*	10, 4.5%	3, 3.2%	0.01
Vaccine of COVID-19				0.000
0 Shot (n,%)	54, 14.1%*	82, 37.3%*	85, 92.4%	
1 Shot (n,%)	15, 3.9%	9, 4.1%	2, 2.2%	
2 Shots (n,%)	131, 34.1%*	56, 25.5%*	3, 3.2%	
>=3 Shots (n,%)	184, 47.9%*	73, 33.1%*	2, 2.2%	
Symptoms				
Fever (n, %)	44, 11.4%	29, 13.1%	11, 11.9%	> 0.05
Respiratory symptoms (n,%)	148, 38.5%	92, 41.8%	42, 45.6%	> 0.05
Clinical Classification				0.000
Asymptomatic (n,%)	200, 52.1%*	93, 42.3%*	15, 16.3%	
Mild (n,%)	165, 42.9%	84, 38.2%	32, 34.8%	
Moderate (n,%)	18, 4.7%*	35, 15.9%*	32, 34.8%	
Severe (n,%)	0*	2, 0.9%	3, 3.3%	
Critical (n,%)	1, 0.3%*	6, 2.7%*	10, 10.8%	
Treatment				
Antibiotics (n,%)	4, 1.0%*	8, 3.6%*	24, 26.1%	0.000
Paxlovid (n,%)	24, 6.3%	18, 8.1%	7, 7.6%	> 0.05
Laboratory data				
ALT (iu/L)	21.5(18)^#^	20(16)^#^	13(11.2)	0.000
AST (iu/L)	21(9)^#^	22(9.7)^#^	14(8)	0.000
Albumin (g/L)	41.53 ± 3.02^#^	39.92 ± 4.22^#^	36.94 ± 4.35	0.000
Blood urea nitrogen (mmol/L)	4.40 ± 1.11^#^	5.63 ± 2.03^#^	38.81 ± 13.13	0.000
Creatinine (umol/L)	71.02 ± 12.03^#^	87.35 ± 15.59^#^	1253.6 ± 442.7	0.000
eGFR (ml/min/1.73m^2^)	106.91 ± 13.01^#^	76.07 ± 12.50^#^	3.46 ± 1.83	0.000
Sodium (mmol/L)	139.74 ± 2.30^#^	139.73 ± 3.44^#^	136.43 ± 3.97	0.000
Chloine (mmol/L)	103.39 ± 2.66^#^	103.86 ± 3.06^#^	100.72 ± 5.07	0.000
Potassium (mmol/L)	4.19 ± 0.49^#^	4.16 ± 0.5^#^	5.10 ± 0.93	0.000
Bicarbonate (mmol/L)	26.26 ± 2.34^#^	26.41 ± 2.5^#^	18.35 ± 4.30	0.000
Calcium (mmol/L)	2.30 ± 0.09^#^	2.26 ± 0.10^#^	2.16 ± 0.22	0.000
Phosphorus (mmol/L)	1.20 ± 0.22^#^	1.13 ± 0.22^#^	2.47 ± 1.10	0.000
White blood cell (10^^9^/L)	5.34 ± 1.98	5.45 ± 2.09	5.14 ± 2.59	> 0.05
Neutrophils (10^^9^/L)	3.11 ± 1.71^#^	3.34 ± 1.88*	3.83 ± 2.39	0.004
Hemoglobin (g/L)	141.66 ± 18.37^#^	137.65 ± 20.0^#^	102.59 ± 18.17	0.000
Lymphocyte (10^9/L)	1.69 ± 0.65^#^	1.54 ± 0.63^#^	0.85 ± 0.46	0.000
Monocyte (10^^9^/L)	0.45 ± 0.20	0.48 ± 0.25	0.47 ± 0.26	> 0.05
Platelet (10^^9^/L)	210.18 ± 58.47^#^	190.72 ± 57.80^#^	134.97 ± 52.98	0.000

Group A: eGFR > = 90ml/min/1.73m2, Group B: 15ml/min/1.73m2 < = eGFR < 90ml/min/1.73m2, Group C: Dialysis-dependent patient

CVD:* ALT*: Alanine aminotransferase, *AST *Aspartate aminotransferase, *CVD *Cardiovascular disease, *eGFR *estimated-Glomerular filtration rate

* : Compared with Group C, *P* < 0.05; # : Compared with Group C, *P* < 0.0

### Outcomes of patients with COVID-19

A total of 13 (2.15%) patients were admitted to the ICU, with 8 (61.5%) of them being hemodialysis (HD) patients. HD patients exhibited significantly prolonged durations of hospitalization and positive nucleic acid test compared to the other two groups. Furthermore, dialysis patients had a higher likelihood of progressing to severe cases and experienced a longer recovery period compared to the other groups (Table 2).

**Table 2:** Outcomes of patients with COVID-19

	Group A *n* = 384	Group B *n* = 220	Group C *n* = 92	*P value*
Admission to ICU (n,%)	2, 0.5%*	3, 1.3%*	8, 8.6%	0.000
Duration of hospitalization (d)	13.18 ± 5.10^#^	13.52 ± 5.74^#^	18.49 ± 7.76	0.000
Duration of positive nucleic acid test (d)	10.44 ± 3.77^#^	11.61 ± 4.49^#^	17.25 ± 6.75	0.000

Group A: eGFR > = 90ml/min/1.73m2, Group B: 15ml/min/1.73m2 < = eGFR < 90ml/min/1.73m2, Group C: Dialysis-dependent patient

*ICU *Intensive care unit

*: Compared with Group C, *P* < 0.05; # : Compared with Group C, *P* < 0.01

### Cumulative hazards of admission to ICU in COVID-19 patients with different eGFR

**Fig. 1:**
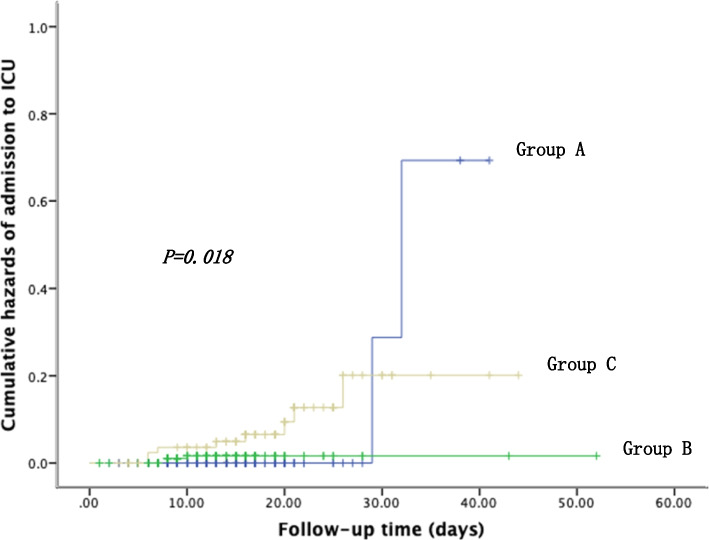
Cumulative hazards of admission to ICU in different groups

At the follow-up endpoint, the incidences of ICU admission in each group were 0.5% (Group A), 1.3% (Group B), and 8.6% (Group C), respectively. The HD group had a significantly higher proportion of severe or critical cases compared to the other two groups (*P* = 0.018). Within a 3-week period, a total of 372 patients in Group A (96.9%), 207 patients in Group B (94.1%), and 61 patients in Group C (66.3%) had been successfully treated and discharged (Fig. 1).

### Influencing factors for the duration of positive nucleic acid test

In the univariate linear regression analysis, several variables commonly associated with worsening infection were included as potential risk factors. These variables included age, sCr, BUN, eGFR, albumin, electrolyte levels, transaminase levels, and blood cell counts (Table 3).

**Table 3:** Univariate linear regression analysis

	*B*	*t*	*P Value*	*95%CI*
Age (y)	0.087	8.496	0.000	0.067 ~ 0.108
Blood urea nitrogen (mmol/L)	0.146	9.828	0.000	0.117 ~ 0.176
Creatinine (umol/L)	0.005	11.017	0.000	0.004 ~ 0.006
eGFR (ml/min/1.73m^2^)	-0.061	-12.616	0.000	-0.071 ~ 0.052
ALT (iu/L)	-0.014	-1.73	0.084	-0.029 ~ 0.002
AST (iu/L)	-0.009	-0.909	0.364	-0.030 ~ 0.011
Albumin (g/L)	-0.32	-6.831	0.000	-0.412~-0.228
Potassium (mmol/L)	1.117	3.847	0.000	0.547 ~ 1.687
Sodium (mmol/L)	-0.281	-4.759	0.000	-0.397~-0.165
Chloine (mmol/L)	-0.174	-3.139	0.002	-0.283~-0.065
Calcium (mmol/L)	-6.116	-4.192	0.000	-8.981~-3.251
Phosphorus (mmol/L)	1.76	5.778	0.000	1.162 ~ 2.357
Bicarbonate (mmol/L)	-0.359	-7.385	0.000	-0.455 ~ 0.264
White blood cell (10^^9^/L)	-0.295	-3.241	0.001	-0.473~-0.116
Hemoglobin (g/L)	-0.062	-7.61	0.000	-0.078 ~ 0.046
Neutrophils (10^^9^/L)	-0.103	-1.006	0.315	-0.305 ~ 0.098
Lymphocyte (10^9/L)	-1.947	-7.27	0.000	-2.473~-1.421
Monocyte (10^^9^/L)	1.333	1.616	0.107	-0.287 ~ 2.953
Platelet (10^^9^/L)	-0.022	-7.672	0.000	-0.028~-0.017

*AST *Aspartate aminotransferase, *CVD *Cardiovascular disease, *eGFR *estimated-Glomerular filtration rate

Variables with a *p*-value less than 0.1 in the univariate linear regression analysis were selected for inclusion in the multiple linear regression analysis. The findings showed the duration of positive nucleic acid test was negatively correlated with eGFR, platelet or lymphocyte counts. Specifically, patients with lower eGFR and lower lymphocyte/platelet counts exhibited a tendency towards prolonged hospitalization (Table 4).

**Table 4:** Multivariate linear regression analysis

	*B*	*t*	*P Value*	*95%CI*
Constant	18.725	29.814	0.000	17.492~-19.958
eGFR (ml/min/1.73m2)	-0.048	-8.462	0.000	-0.059~-0.037
Platelet (10^9/L)	-0.011	-3.388	0.001	-0.017~-0.005
Lymphocyte (10^9/L)	-0.658	-2.259	0.024	-1.229~-0.086

*eGFR *estimated-Glomerular filtration rate

## Discussion

### Key results and interpretation

Despite the Omicron variant is more infectious than any other previous VOC, its morbidity and mortality seems to diminish markedly [9]. Numerous of countries had loosen or completely cancelled previous public health approaches, but the fact told us that we had significantly underestimated the infectivity of this new variant. Since the discovery of the omicron variant, 2152 omicron cases had been identified from 57 countries just within 16 days (November 24 ~ December 9, 2021) [10]. By March 2022, COVID-19 pandemic was mainly caused by the Omicron BA.2 variant [3]. Due to the surge in infections, the deaths also increased again significantly [3]. From March 2022 to May 2022, the BA.2 variant caused a total of nearly 650,000 infections and approximately 600 deaths during this pandemic in Shanghai [4]. In the light of this, we aim to describe the clinical characteristics and outcome in 696 COVID-19 patients with different renal functions statuses during the dominant prevalence of Omicron BA.2 in Shanghai.

Prior to the outbreak of this pandemic, free COVID-19 vaccinations had been widely implemented throughout China. As a result, 444 (73.5%) non-dialysis patients had received at least two doses of the COVID-19 vaccine, while 85 (92%) HD patients didn’t receive any COVID-19 vaccination. Due to worry about the possible side effects caused by the vaccine, most of the ESRD patients refused the COVID-19 vaccination. Even in the United States, many HD patients also hesitated to receive a vaccination for the same reason [11]. Given the comorbidities, impaired immunity, and frequently gathering in dialysis centers, HD patients are at a greater risk of COVID-19 infection. Therefore, the three major nephrology societies including the Internal Society of Nephrology, the American Society of Nephrology, and European Dialysis and Transplant Association—European Renal Association all recommended COVID-19 vaccine priority for dialysis patients [12–14]. Through this pandemic, we found a significantly higher infection and severity rate of COVID-19 in HD patients compared to the other patients, so we should try to encourage dialysis patients to receive early vaccination.

The ZOE COVID study [15] reported that among those testing positive when omicron was dominant, the most frequent symptoms from 4990 cases were runny nose (76.5%), sore throat (70.5%), sneezing (63%) ,cough (49.8%), hoarse voice (42.6%), and fever (> 30%). In contrast, the patients in our study exhibited relatively mild symptoms. Among them, 308 cases (44.2%) were asymptomatic, only 84 cases (12.1%) were fever, and 282 cases (40.5%) had respiratory symptoms such as cough, sore throat, chest pain, shortness of breath, hoarseness, and hemoptysis. Owing to the city-wide screening in Shanghai during this pandemic, COVID-19 patients can be diagnosed so rapidly even before symptom onset. Additionally, the extensive implementation of COVID-19 vaccinations across China prior to this epidemic has likely contributed to the milder symptomatology observed in these patients.

As anticipated, CKD had been confirmed as one of most common and strongest risk factors for severe COVID-19 [6, 16]. A nationwide analysis from Turkey [17] revealed that the ICU admission rate of COVID-19 patients without CKD (8%) was considerably lower than those of CKD group (39.4%) or HD group (25.4%). In our study, we observed a higher number of severe/critical cases, ICU admission rates, and inpatient days in the HD group than in the other two groups. CKD has emerged as the highest risk for severe COVID-19. And the risk increases as the eGFR decreases, with the highest risk in end stage renal disease patients. Although CKD patients are more susceptible to severe infection, the precise reasons why they are greater vulnerability for severe COVID-19 should be further explored. Such investigations will provide valuable insights for establishing treatment measures for this population.

In addition to the eGFR, we found that the nucleic acid positive days was also negatively correlated with lymphocyte and platelet counts through the multiple regression analysis. In COVID-19 patients, the total blood lymphocyte count, especially that of T cells, is lower than in healthy controls [18]. Lymphopenia is more severe in symptomatic compared with asymptomatic ones. Moreover, both CD4 + and CD8 + T cell blood counts are further decreased in severe cases compared with moderate cases. In elderly COVID-19 patients, high lymphocyte count was predictive of a better outcome [19]. The humoral response to SARS-CoV-2 infection is ubiquitous among infected individuals. Not only the B cell and plasma cell are responsible for producing antibodies, but also the circulating virus-specific CD4 + and CD8 + T cell responses strongly correlated with the IgG titers. So lymphocytes, including B cells and T cells, are the key components of the human immune system’s response to SARS-CoV-2.

A meta-analysis on 7613 COVID-19 patients reported a significant relationship between thrombocytopenia and the clinical severity of COVID-19 [20]. Reduction of platelet count was identified as a poor prognostic factor. Although the decrease of platelet number is multifactorial, in the context of COVID-19 it may be mainly due to decreased production of platelets in damaged lungs [21]. Alternatively, an increased platelet clearance maybe the result of the activation of the immune system that induces an antibody-mediated phagocytic response [22]. Lastly, another hypothesis is that microthrombi formation cause the increased consumption of platelets [21].

### Limitations

There were several limitations to this study. Firstly, it is important to acknowledge that this is a single-center retrospective study with a relatively small sample size, which may limit the generalizability of the findings. The absence of a national or citywide COVID-19 database system prevented us from accessing comprehensive data beyond the scope of this study. Secondly, the follow-up time of this study was limited to the length of hospitalization, so the long-term outcomes of COVID-19 could not be observed. Additionally, the unavailability of urine samples hindered us to determine whether the patients with GFR > = 60ml/min/1.73m^2^ belonged to CKD population. Another limitation was that no formal power analysis was carried out to determine the appropriate sample size. The recruitment of patients was limited to a specific subset of hospitalized individuals in our hospital, potentially introducing selection bias.

### Generalizability

In conclusion, the prognosis for the majority of individuals with the Omicron variant is favorable. It appears that only HD patients have poorer outcomes and a higher rate of ICU admission. Given the situation of pretty low vaccination rates in HD patients, we should encourage more HD patients to receive COVID-19 vaccination. Decreased GFR, low lymphocyte/platelet counts serve as significant risk factors for prolonged hospitalization. Therefore, more attention should be directed towards these patients to improve their clinical outcomes.

## Data Availability

The datasets used and/or analyzed during the current study are available from the corresponding author upon reasonable request.
